# Rules-based Volumetric Segmentation of Multiparametric MRI for Response Assessment in Recurrent High-Grade Glioma

**DOI:** 10.21203/rs.3.rs-3318286/v1

**Published:** 2023-09-11

**Authors:** Harshan Ravi, Samuel H. Hawkins, Olya Stringfield, Malesa Pereira, Dung-Tsa Chen, Heiko Enderling, Hsiang-Hsuan Michael Yu, John A. Arrington, Solmaz Sahebjam, Natarajan Raghunand

**Affiliations:** Moffitt Cancer Center; Moffitt Cancer Center; Moffitt Cancer Center; Moffitt Cancer Center; Moffitt Cancer Center; Moffitt Cancer Center; Moffitt Cancer Center; Moffitt Cancer Center; Moffitt Cancer Center; Moffitt Cancer Center

**Keywords:** recurrent high-grade glioma, tumor heterogeneity, tumor volumetrics, magnetic resonance imaging, pixel intensity calibration

## Abstract

We report domain knowledge-based rules for assigning voxels in brain multiparametric MRI (mpMRI) to distinct tissuetypes based on their appearance on Apparent Diffusion Coefficient of water (ADC) maps, T1-weighted unenhanced and contrast-enhanced, T2-weighted, and Fluid-Attenuated Inversion Recovery images. The development dataset comprised mpMRI of 18 participants with preoperative high-grade glioma (HGG), recurrent HGG (rHGG), and brain metastases. External validation was performed on mpMRI of 235 HGG participants in the BraTS 2020 training dataset. The treatment dataset comprised serial mpMRI of 32 participants (total 231 scan dates) in a clinical trial of immunoradiotherapy in rHGG (NCT02313272). Pixel intensity-based rules for segmenting contrast-enhancing tumor (CE), hemorrhage, Fluid, non-enhancing tumor (Edema1), and leukoaraiosis (Edema2) were identified on calibrated, co-registered mpMRI images in the development dataset. On validation, rule-based CE and High FLAIR (Edema1 + Edema2) volumes were significantly correlated with ground truth volumes of enhancing tumor (R = 0.85;p < 0.001) and peritumoral edema (R = 0.87;p < 0.001), respectively. In the treatment dataset, a model combining time-on-treatment and rule-based volumes of CE and intratumoral Fluid was 82.5% accurate for predicting progression within 30 days of the scan date. An explainable decision tree applied to brain mpMRI yields validated, consistent, intratumoral tissuetype volumes suitable for quantitative response assessment in clinical trials of rHGG.

## Introduction

As complex multimodality therapeutic regimens are developed and investigated for glioblastoma (GBM), the accurate and timely imaging diagnosis of disease progression becomes more challenging for the neuroradiologist and neuro-oncologist. In 2010, the Response Assessment for Neuro-Oncology (RANO) working group introduced guidelines under which high-grade glioma (HGG) is classified as progressive if the sum of the product of bidimensional tumor diameter on contrast-enhancing (CE) MRI increases by 25%, there is a significant increase in non-enhancing (NE) tumor, the appearance of new lesions, or clinical deterioration^1^. Subsequently, immunotherapy RANO (iRANO) criteria were introduced for evaluating confounding MRI findings that meet RANO criteria for progression within the first 6 months of initiating immunotherapy. iRANO recommends continuing the therapy until disease progression is confirmed on a 3-month follow-up scan or upon a significant clinical decline unrelated to the comorbid event^2^.

While RANO remains the gold standard for response assessment in HGG, there is significant inter-observer variability in estimating tumor size using 2-dimensional (2D) measurements, with a need for improved differential identification of NE tumor from co-existing areas of vasogenic edema and gliosis on Fluid-Attenuated Inversion Recovery (FLAIR) images^3^. Volumetric assessment has been proposed as a complement to standard 2D assessment. For example, in a phase II trial (RTOG 0625/ACRIN 6677) in recurrent HGG (rHGG), Boxerman et al. found that early contrast-enhanced T1-weighted (T1wCE) volumes were a significant prognostic marker for overall survival (OS)^4^. In a multicenter phase III trial (ACRIN 6686/RTOG 0825) of newly-diagnosed GBM, Boxerman et al. found that FLAIR adds value beyond bidimensional RANO measurements in predicting OS^5^. In recurrent GBM treated with bevacizumab, Ellingson et al. found that the pre-treatment ratio of FLAIR to CE volume was predictive of progression-free survival (PFS) and OS, while post-treatment CE volume by itself was predictive of PFS^6^.

However, manual delineation of disease-related volumes is time-intensive and laborious, with significant inter-rater variability, while standard image segmentation algorithms are insufficient to handle significant spatial heterogeneity such as in GBM. The Brain Tumor image Segmentation benchmark (BraTS) challenges have made available an extensive repository of multisite standard-of-care (SOC) multiparametric MRI (mpMRI) images of preoperative GBM along with expert manual annotations of peritumoral edema (ED), contrast-enhancing tumor (ET), and the necrotic and non-enhancing tumor core (NCR/NET) sub-regions^7–9^. Machine learning-driven tools resulting from successive BraTS challenges and the multisite federated learning consortium^10^ are showing promise for automated delineation of the surgically actionable mass in preoperative GBM.

**Figure 1:**
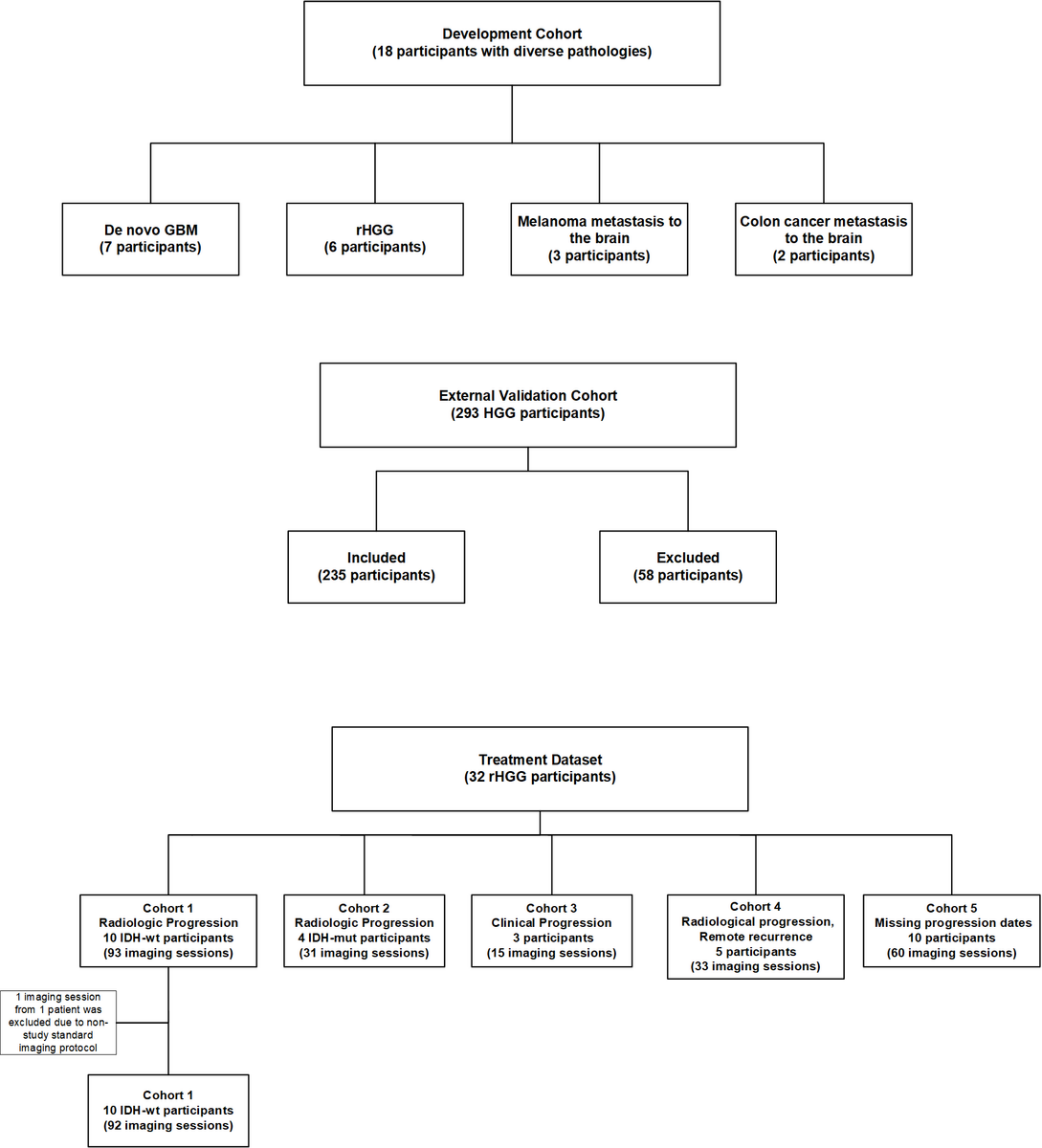
Imaging datasets in this study.

The SOC in newly-diagnosed GBM involves maximal surgical resection, followed by radiotherapy plus concomitant and maintenance temozolomide^11^. New therapeutics for GBM are tested primarily in recurrent disease, for which the segmentation tools developed on mpMRI of preoperative GBM do not perform adequately. Challenges include the presence of a surgical resection cavity and the fact that rHGG need not present as a single, well-defined mass. The latter characteristic is also a challenge for human experts who are tasked with manually contouring tumor masses in rHGG to provide ground truth for training deep learning models. There is a need for automated methods to quantify tumor volume to support clinical trials in rHGG. To address this need, we developed domain knowledge-based rules to assign brain voxels to distinct tissuetypes based on their appearance on co-registered mpMRI images of 18 subjects with pre-operative GBM, rHGG, and brain metastases. Volumes of intratumoral tissuetypes computed using these rules were externally validated against ground truth volumes of ET, ED, and NCR/NET tissuetypes in 235 HGG participants from the BraTS 2020 training dataset. Finally, the consistency and utility of intratumoral tissuetype volumes computed per patient using these rules was demonstrated in a retrospective analysis of serial mpMRI collected in a clinical trial of hypofractionated reirradiation, bevacizumab, and pembrolizumab to treat rHGG (NCT02313272, 32 participants, total 231 scan dates)^12^. The imaging datasets used in this study are summarized in Fig. 1.

## Results

### Qualitative Analysis of Tissuetype Segmentation Maps in the Development Cohort.

**Figure 2:**
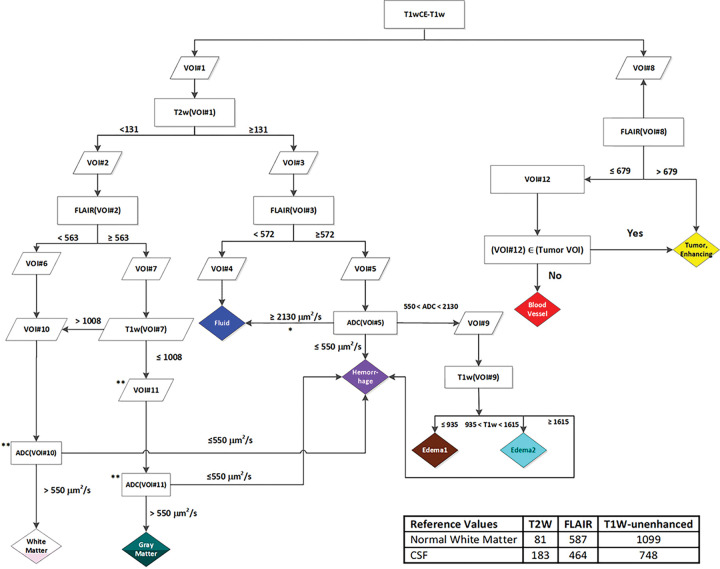
Decision tree for classifying voxels in calibrated co-registered multiparametric (mpMRI) images into tissuetypes. Box colors match the color scheme used in voxelwise tissuetype maps. Reference values used for calibration are shown in the inset table. *This step was omitted during analysis of BraTS 2020 data, which did not include ADC maps. **These steps were skipped to reach the next step in the flow chart when analyzing BraTS 2020 data.

**Figure 3:**
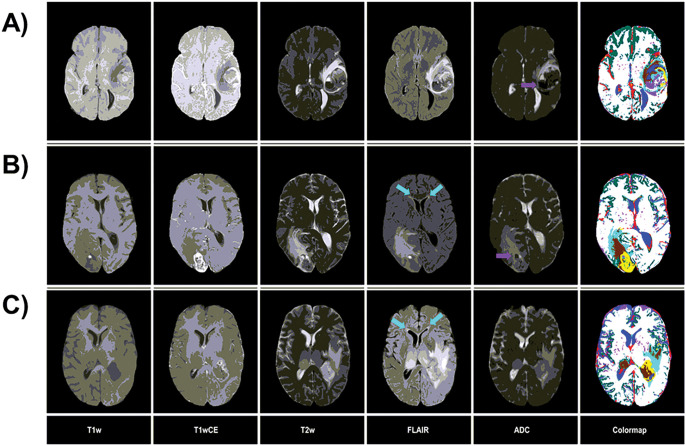
Examples of tissuetype segmentation colormaps from the development dataset. A) Melanoma, B) Colon cancer, and, C) Glioblastoma (GBM). In each row, from left to right, the first 5 images are multiparametric (mpMRI), and the final image is the tissuetype segmentation (colormap). The labels at the bottom indicate the mpMRI image type. Purple and cyan arrows indicate areas of hemorrhage and areas with suspected leukoaraiosisrespectively. The colormap legend is as follows: red = Blood Vessel, yellow = CE tumor, brown = Edema1, cyan = Edema2, purple = Hemorrhage, blue = Fluid, dark green = Gray matter, off-white = White matter.

As described in detail in the Materials and Methods section, the thresholds in Figure 2 were iteratively optimized to segment Fluid (blue) and vasculature (red) in non-tumor areas, and CE (yellow) and NE tumor within the tumor VOI, to match Radiologist (JAA) expectations based on the T1-weighted unenhanced (T1w) and contrast-enhanced (T1wCE), T2-weighted (T2w), FLAIR, and ADC images. Thresholds were also selected for segmenting GM (green) and WM (white), though the mpMRI acquisitions were not optimized for this task. Figure 3 illustrates tissuetype maps (“colormap”) computed from co-registered mpMRI images of participants with melanoma (Figure 3A) and colon cancer (Figure 3B) metastases to the brain, and GBM (Figure 3C). It can be seen that Edema2 (cyan) co-localizes with regions of leukoaraiosis (arrows) (magnified view shown in supplementary figure S2), while significant portions of the high FLAIR regions within the tumor VOI are classified as Edema1 (brown). The rules correctly identify hemorrhage (purple) in melanoma and colon metastases (Figures 3A & 3B) but also artifactually label edge pixels affected by EPI distortions in the ADC map (Figure 3C).

### Validation of Tissuetype Volumes vs. Ground Truth in an External Cohort.

**Figure 4:**
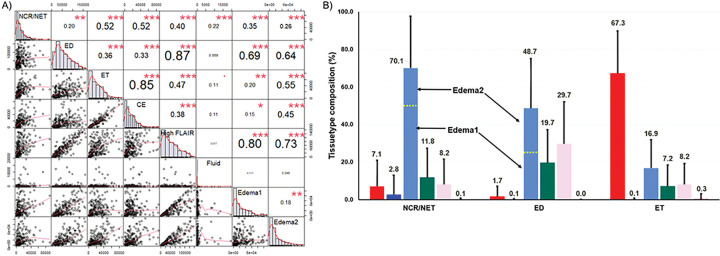
Validation of tissuetype volumes against ground truth tumor segmentations in the BraTS 2020 data. (A) A correlation matrix with pairwise scatterplots of 3 ground truth BraTS 2020 segmentations and 5 tissuetypes (lower half), and corresponding pairwise correlation values (upper half) are shown. The diagonal elements show the distribution of values in each segmentation and tissuetype. The symbols (“***”, “**”, “*”, “.”, “ ”) correspond to p-values of (0.001, 0.01, 0.05, 0.1, 1), respectively. (B) The tissuetype composition of each BraTS segmentation is shown. The dotted yellow line separates the contribution of Edema1 and Edema2 to the High FLAIR tissuetype. The tissuetype composition values in percentage are indicated on top of each bar.

Segmentation methods are typically evaluated on the basis of Dice score or equivalent voxelwise similarity with a ground truth. However, our primary purpose is to perform volumetrics of intratumoral tissuetypes. We have therefore compared the volumes of specific tissuetypes computed using the algorithm in Figure 2 to manually segmented volumes of ET, ED, and NCR in the BraTS 2020 dataset. CE tissuetype computed using the algorithm in Figure 2 was strongly correlated with BraTS ET (R=0.85; p<0.001; Figure 4A). On average, the manually drawn ET primarily encompassed CE voxels (~67%) with smaller contributions from other tissuetypes (Figure 4B). The BraTS ED component was highly correlated with High FLAIR (R=0.87; P<0.001) and moderately correlated with Edema1 (R=0.69; P<0.001) and Edema2 (R=0.64; P<0.001) individually (Figure 4A). The manually drawn ED encompassed pixels classified as High FLAIR (49%) and normal brain tissue (49%) (Figure 4B). Unlike ET and ED, NCR/NET was weakly correlated with CE, High FLAIR, intratumoral Fluid, Edema1, and Edema2 (R=0.22–0.52, Figure 4A).

**Figure 5:**
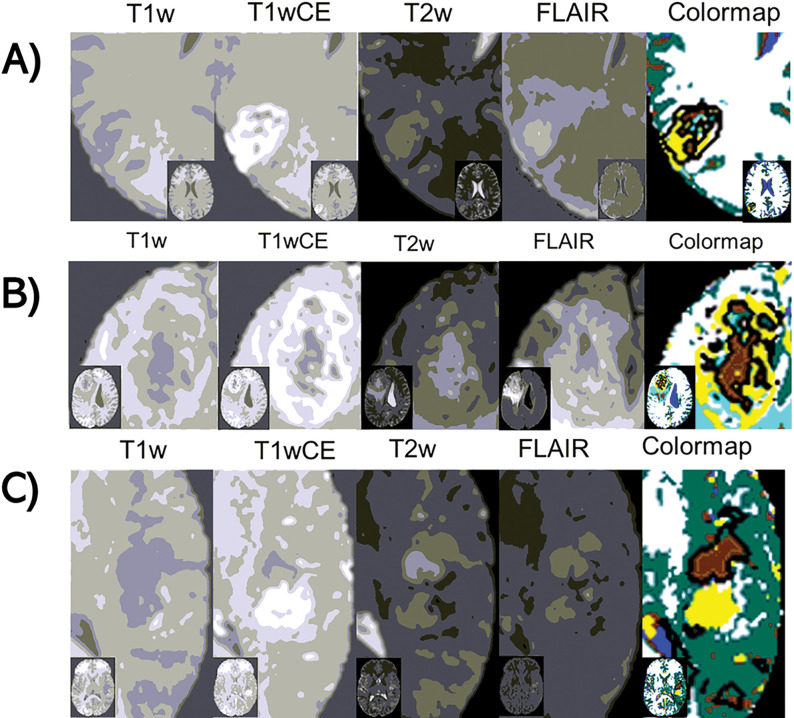
Comparisons of tissuetype colormaps with ground truth segmentations in illustrative examples from the BraTS 2020 data. Ground truth segmentations of A) Enhancing tumor (ET), B) Necrotic Core/Non Enhancing tumor (NCR/NET), and C) Peritumoral Edema (ED) are overlaid on the tissuetype colormap. The colormap legend is as follows: red = Blood Vessel, yellow = CE tumor, brown = Edema1, cyan = Edema2, purple = Hemorrhage, blue = Fluid, dark green = Gray matter, off-white = White matter.

Figure 5 depicts overlays of ground truth BraTS segmentations of NCR/NET, ED, and ET onto tissuetype maps in illustrative examples. Qualitatively, there is good agreement between manually drawn ET and voxelwise CE, and between manually drawn ED and voxelwise Edema1 and Edema2 tissuetypes. It is also apparent that the manually drawn ET, ED, and NCR/NET segmentations encompass some voxels with divergent tissuetype signatures.

### Univariable Correlations with TTP in Treatment Dataset.

**Figure 6:**
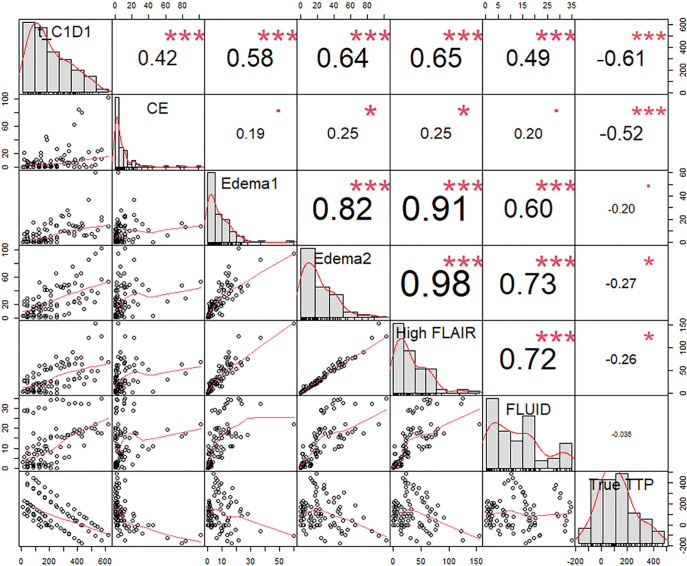
A correlation matrix of 5 tissuetypes and number of days elapsed since day 1 of cycle 1 of treatment (t_C1D1) (independent variables), and time-to-progression (TTP) (dependent variable), in the treatment dataset. The pairwise scatterplots are shown in the lower half, with the corresponding pairwise correlation values in the upper half. The diagonal elements show the distribution of values in each variable in the data. The symbols (“***”, “**”, “*”, “.”, “ ”) correspond to p-values of (0.001, 0.01, 0.05, 0.1, 1), respectively.

Figure 6 presents the cross-correlations of CE, High FLAIR, Edema1, Edema2, intratumoral Fluid, t_C1D1, and TTP. On a univariable basis, t_C1D1 was strongly correlated with TTP (R=−0.61), followed by CE (R=−0.52), Edema2 (R=−0.27), High FLAIR (R=−0.26), Edema1 (R=−0.20), Fluid (R=−0.036). Edema1, Edema2, and High FLAIR were strongly correlated with each other (R=0.82–0.98) and moderately correlated with t_C1D1 (R=0.58–0.65). Intratumoral Fluid was moderately correlated with Edema1, Edema2, and High FLAIR (R=0.60–0.73) and weakly correlated with t_C1D1 (R=0.49) and CE (R=0.20).

### Tissuetype Volume Trends in Treatment Dataset.

**Figure 7:**
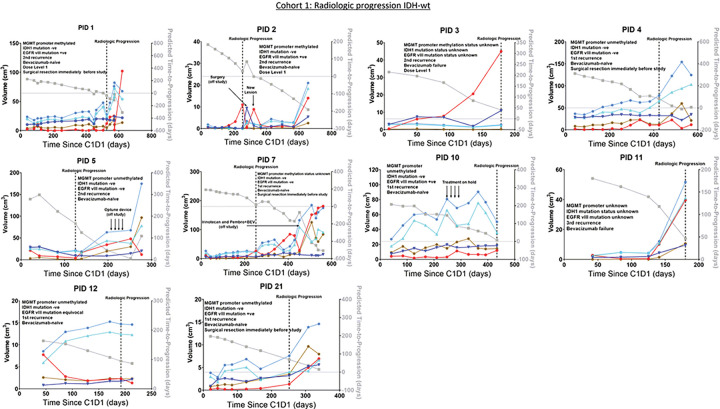
Temporal dynamics of tissuetype volumes within the abnormal volume of interest (abVOI) (left Y-axis, in cm^3^), and the model-predicted TTP (right Y-axis, in days), for participants in the treatment dataset with isocitrate dehydrogenase (IDH) wild type (IDH-wt) tumors and radiologic progression (Cohort 1). Tissuetype volumes plotted on the left Y-axis are: red = CE tumor, brown = Edema1, cyan = Edema2, blue = Fluid, and light blue = High FLAIR (= Edema1 + Edema2). The gray line and symbols show the per-timepoint predictions of time-to-progression (TTP; right Y-axis). The vertical dashed line indicates the recorded progression day. Relevant clinical details for each participant are listed on each plot. Equivalent data from participants in Cohorts 2–5 are presented in supplementary figures S3-S6.

Temporal dynamics of the volumes of CE (red), Fluid (blue), High FLAIR (light blue), Edema1 (brown), and Edema2 (cyan) tissuetypes within the abVOI are presented in figure 7 for 10 treatment study participants. In 7 of 10 participants in Cohort 1 (figure 7), there was an increasing trend in the CE tissuetype volume beginning an average of 100 days before progression was called. An inconsistent trend in the volumes of Edema1, Edema2, High FLAIR, and Fluid was observed in the majority of participants in Cohort 1. Intratumoral tissuetype volume dynamics for 22 treatment study participants in Cohorts 2–5 are presented in Supplementary Figures S3-S6. Over the majority of 231 scan dates of the 32 participants in the treatment dataset, the timepoint to timepoint changes in volumes of all intratumoral tissuetypes are qualitatively smooth, lending confidence that application of the rules in Figure 2 to intensity-calibrated mpMRI yields consistent volumetric data that can meaningfully inform longitudinal assessment of rHGG.

### Multivariable Model for Predicting TTP in Treatment Dataset.

To further characterize the utility of the proposed mpMRI tissuetyping algorithm in a clinical trial setting, we undertook a proof-of-concept multivariable analysis to predict TTP from t_C1D1 and the volumes of CE, High FLAIR, Edema1, Edema2, and Fluid within the abVOI. Across 92 time points in Cohort 1, we found significant relationships between TTP and t_C1D1 (p<1.80×10^−10^), CE (p<1.89×10^−4^), and Fluid (p<8.15×10^−5^). All three variables statistically significantly predicted TTP (F(3,88)=34.73, p < 6.643×10^−15^, Multiple R^2^=0.54, Adjusted R^2^=0.53), and a model combining them yielded the lowest AIC with 10-fold cross-validation:

(1) TTP=205.6−0.6•(t_C1D1)−2.5•(CE)+4.8•(Fluid)

Where TTP and t_C1D1 are in units of days, and CE and Fluid are in cm^3^.

### Model Accuracy for Predicting Progression in Treatment Dataset.

The model-predicted TTP (plotted in gray) was generally in good agreement with the retrospectively-adjusted RANO-based progression^12^ (vertical dashed line) for most participants in Cohort 1 with IDH-wt tumors (Figure 7). Model performance is qualitatively lower on participants with IDH-mutant tumors (Cohort 2, supplementary figure S3), participants with clinical progression (Cohort 3, supplementary figure S4), and participants with remote recurrence (Cohort 4, supplementary figure S5). The model in equation (1) is a multiple linear regression model that was fitted to continuous TTP data from 92 time points, and we also investigated its performance for predicting binary outcomes. Model accuracy for predicting if progression would happen within n days of a given scan date is summarized in Table 3 for Cohorts 1–4. In the radiological progression Cohorts 1, 2, and 4, model accuracy was 80–88.5% for predicting events 30 days in the future from the scan date, decreasing to 40–73.7% for predicting events 90 days after the scan date. Expectedly, model performance for each value of n was worse in participants with clinical progression (Cohort 3) than when progression was on local radiologic changes (Cohorts 1 and 2).

## Discussion

Tumor volumetrics can meaningfully augment response assessment in GBM^3–6^. Manual segmentation of disease-associated volumes is arduous, and classical automatic segmentation algorithms are inadequate for delineating heterogeneous tumors. Collaborative initiatives such as the BraTS challenges^7–9^ and FETS^10^ have yielded deep learning-driven tools for segmenting preoperative GBM, but these do not perform adequately for segmenting rHGG. To address this need, we present explainable rules for combining information from co-registered SOC mpMRI and ADC images to segment HGG into distinct intratumoral tissuetypes. The algebraic manipulations required to calibrate images and compute tissuetypes by this method are based on domain knowledge and easy to understand compared with “black box” deep learning models. These voxelwise rules are unaffected by the presence of resection cavities and therefore equally applicable to preoperative GBM and rHGG, arguably making them more generalizable than deep learning approaches.

In a diverse external validation dataset, volumes of intratumoral CE and High FLAIR tissuetypes computed using the proposed rules are significantly in agreement with ground truth volumes of ET and ED, respectively. While concordance at the cohort level between rules-based tissuetype volumes and BraTS 2020 ground truth is reassuring, the computed volumes of mpMRI tissuetypes also need to be consistent from scan date to scan date at the level of the individual patient for the approach to be useful in clinical trials of new therapeutics in rHGG. We applied the proposed rules to serial mpMRI images collected in a clinical trial of rHGG treated with hypofractionated reirradiation, bevacizumab and pembrolizumab, for which we have described clinical results previously^12^. Timepoint to timepoint changes in the volumes of CE, Fluid, High FLAIR, Edema1, and Edema2 tissuetypes within the abVOI are qualitatively smooth in 32 tumors in this treatment dataset, which demonstrates the intra-patient consistency of tissuetype volumetrics using the proposed method. On training data from 10 subjects (92 scan dates) in the treatment dataset, a model that combined per-timepoint volumes of CE and intratumoral Fluid tissuetypes and t_C1D1 was 82.5% accurate for predicting whether progression would occur within 30 days of a given scan date. Though the TTP prediction model (Eq. 1) itself requires validation on an independent treatment dataset, the statistically significant correlations between individual tissuetype volumes and TTP are quantitative evidence that the proposed method for intratumoral tissuetype volumetrics is consistent and reproducible across multiple rHGG subjects and scan dates.

We utilized an intensity-calibration method that we have described previously^13^ to compensate for the fact that pixel values in MRI are in arbitrary units and not comparable across imaging sessions or scanners or image acquisition parameter variations. We demonstrated the generalizability of our rules-based tissuetype segmentations of calibrated images against ground truth segmentations provided with external BraTS data that were acquired at multiple institutions on a mix of 1.5 T and 3 T scanners from multiple institutions. Rules-based volumes of CE and High FLAIR were in good agreement with BraTS segmentations of ET (R = 0.85) and ED (R = 0.87), respectively. BraTS NET/NCR was only moderately correlated with rules-based tissuetypes (R = 0.22–0.52) despite manually-drawn NCR/NET contours encompassing primarily High FLAIR (70%) tissuetype, possibly because a significant portion of the uncorrelated High FLAIR component came from regions within the tumor VOI that lay outside the NCR/NET contour. By contrast, rules-based High FLAIR tissuetype makes up only ~ 49% of ED but the two are highly correlated (R = 0.87), likely because the tumor VOI has a large overlap with the manually-drawn ED contour. ADC-related rules in our algorithm were ignored during validation due to the absence of ADC maps in BraTS data. ADC rules are crucially required to identify fluid within resection cavities, and regions of hemorrhage. BraTS 2020 data are limited to pre-surgical GBM, so this modification did not significantly affect the validation results.

Temporal dynamics of mpMRI signatures of intratumoral heterogeneity in GBM can inform response assessment at each scan date. For example, Ananthnarayan et al.^14^ showed that the edema-to-tumor volume ratio in GBM followed a different temporal trend and was 38.4% lower at progression after extended bevacizumab treatment vs. after chemotherapy. Tran et al.^15^ found that the FLAIR hyperintensity volume increased at a lower rate in IDH-mut tumors than in IDH-wt tumors after radiochemotherapy, while the T1wCE volume exhibited the opposite behavior. Song et al.^16^ found that interval change of relative ADC within enhancing tumor was promising for discriminating treatment effects from progression in recurrent GBM within the first 6 months following immune checkpoint inhibition. Manually-delineated contrast-enhancing tumor volumes from serial T1wCE showed promise for predicting tumor trajectory in a subset of patients in NCT02313272^17^. In our proof-of-concept demonstration, single time point volumes of CE and Fluid tissuetypes within a loosely drawn tumor VOI, in combination with t_C1D1, were predictive of TTP in rHGG treated with immunoradiotherapy. TTP decreased by 0.59% ± 0.08% and 2.49% ± 0.64% for every 1% increase in respective t_C1D1 and CE, and increased by 4.79% ± 1.16% for every 1% increase in intratumoral Fluid. The negative correlation between CE volume within the abVOI and TTP is unsurprising given RANO definitions of progression, while the negative correlation between t_C1D1 and TTP reflects the inevitability of progression becoming more likely with time. The weak positive correlation between Fluid volume within the abVOI and TTP is intriguing, and may reflect the effects of treatment-related cell death^18^, tumor necrosis^19^, and the completeness of surgical resection. High FLAIR, Edema1, and Edema2 were not independently predictive of TTP, possibly due to divergent influences of tumor growth and bevacizumab on the FLAIR signal.

Our study has a few limitations. Firstly, while our rules-based tissuetype segmentation algorithm performed well on external validation, any pixelwise approach is vulnerable to misregistration between the mpMRI sequences. Acquisition of high resolution mpMRI images, and use of robust image registration software, can mitigate this limitation. Secondly, we must consider potential limitations of BraTS segmentations as the ground truth, both due to the challenge of manually tracing highly tortuous image features and the limited precision of any image annotation tool available to the expert annotators. Thus, discrepancies between ET vs. CE, and ED vs. High FLAIR, might be due to deficiencies in either our rules-based approach or in the ground truth segmentations. Thirdly, our rHGG treatment cohort sample size of 32 is small for developing a generalized TTP prediction model, compounded by the availability of progression dates in only 22 study participants. We employed 10-fold cross-validation to mitigate overfitting and identified a parsimonious model with 4 parameters to describe data from 92 time points in Cohort 1, which ensures some degree of generalizability of the model in Eq. (1). Nonetheless, the chief significance of our results in the Treatment Dataset is the demonstration of timepoint to timepoint intra-patient consistency of intratumoral tissuetype volumes computed from the 231 scan dates across 32 study participants. Generalizability of the proof-of-concept TTP prediction model, particularly to rHGG treated with regimens other than reirradiation combined with pembrolizumab and bevacizumab, remains to be investigated on independent test data.

In summary, we report an explainable algorithm for computing volumes of intratumoral tissuetypes from mpMRI of GBM that is easy to understand and applicable to both preoperative and recurrent HGG. Deep learning-based segmentation models are inflexible in that they can only accept the types of input images on which they were trained; a new model must be trained from scratch to exploit information from additional sequences. In contrast, the proposed decision tree is modular, and the basic set of rules for segmentation of co-registered standard mpMRI images can readily be augmented with additional rules to incorporate information from other sequences that may be collected in clinical trials, such as DSC MRI and IVIM. The requisite algebraic manipulations for intensity calibration and voxelwise tissuetyping can conceivably be implemented into a clinical workflow to support new therapeutic studies in rHGG and to enrich the information available to Neuro-Oncology Multidisciplinary Tumor Boards.

## Methods and Materials

### MRI Data Acquisition:

Retrospective analysis of mpMRI images from the cohorts summarized in Fig. 1 was approved by the Moffitt Cancer Center institutional review board. The investigational treatment and imaging for the single-site study NCT02313272 was approved by the Moffitt Cancer Center institutional review board, for which all treatment study participants (Table 1) provided written informed consent as described previously^12^. All human subjects research was conducted in accordance with relevant institutional and national guidelines. mpMRI data in the Development Cohort came from clinical cases chosen to include images of participants with GBM and rHGG (13 participants) and metastases to the brain (melanoma – 3 participants; colon cancer – 2 participants); this mix of patients was chosen to enable the development of generalized intensity-based tissuetype segmentation rules that could accommodate a diversity of pathologies seen on standard clinical MRI of brain tumors, such as hemorrhage, and the varied FLAIR appearances of GBM, metastatic lesions, and leukoaraiosis. BraTS 2020 training data were downloaded from a shared repository (https://ipp.cbica.upenn.edu/). mpMRI data in the Treatment Dataset came from a single-arm, open-label phase I study (NCT02313272) conducted between 2015 and 2019, which enrolled 32 subjects with either bevacizumab naïve or bevacizumab resistant rHGG for treatment with hypofractionated reirradiation (6 Gy x 5 fractions) combined with pembrolizumab (100 or 200 mg, given intravenously every 3 weeks) and bevacizumab (10 mg/kg, given intravenously every 2 weeks), as described previously^12^. Treatment study participants (Table 1) were imaged approximately every 4 to 6 weeks on Siemens 3 T or 1.5 T scanners (Table 2) with a variable number of follow-up scan dates. T1-weighted unenhanced (T1w) and contrast-enhanced (T1wCE), T2-weighted (T2w), FLAIR, and diffusion-weighted images (DWI) were acquired at multiple time points per patient. Apparent diffusion coefficient of water (ADC) maps were computed from DWI by monoexponential fitting.

### MRI Preprocessing

ADC, FLAIR, T1w, and T1wCE were co-registered to the T2w images using in-house MATLAB^®^ code that also resampled the images to match pixel dimensions and slice thicknesses with the reference T2w images. A combination of rigid and affine transformations was used for the spatial co-registration. In only the Treatment Dataset, intra-session co-registration was followed by inter-session co-registration as follows. First, the T2w images from each follow-up visit of a given patient were registered to baseline T2w images of that patient. Then, the resulting transformation computed for each session was applied to the co-registered ADC, FLAIR, T1w, and T1wCE images from that session. To make pixel values in a given scan type comparable across patients and scan dates, co-registered T2w, FLAIR, T1w, and T1wCE images from all datasets were intensity-calibrated using two reference tissues following the method we have described previously^13^.

### Classification of Brain MRI Voxels into Tissuetypes, Algorithm Development:

A decision tree was created based on established knowledge of the relative appearances of multiple normal and pathologic tissuetypes on T1w, T1wCE, T2w, FLAIR, and ADC. Calibrated intensity thresholds at each decision node were adjusted, followed by visual inspection of the resulting tissuetype maps by a neuroradiologist (JAA), to iteratively converge on the final values in Fig. 2 as per the following logic. We first segmented the difference map of T1wCE and T1w into contrast-enhancing voxels (VOI8) and non-enhancing (VOI1) volumes using the MATLAB Otsu thresholding algorithm^20^. Voxels within VOI8 were further sub-classified into contrast-enhancing tumor (CE) and blood vessel (BV) tissuetypes based on differences in calibrated FLAIR MRI intensity. Normal Fluid and pathologic areas of the brain are relatively hyperintense on T2w. Voxels within VOI1 were therefore segmented on calibrated T2w intensity into (i) a hypointense VOI2 containing normal gray matter (GM) and white matter (WM), and, (ii) a hyperintense VOI3 containing Fluid and pathologic tissuetypes. Voxels within VOI2 were further classified as GM or WM based on calibrated FLAIR intensity (WM darker than GM) and calibrated T1w intensity (WM brighter than GM). Additionally, voxels with very low ADC (≤ 550 μm^2^ s^−1^) were classified as hemorrhage. Voxels within VOI3 were classified as Fluid based on hypointensity on calibrated FLAIR or high ADC (≥ 2130 μm^2^ s^−1^), as hemorrhage based on either very low ADC (≤ 550 μm^2^ s^−1^) or hyperintensity on calibrated T1w, with the remainder being classified as edematous tissue. Cutoff values of ADC were taken from the literature for Fluid and WM^21^, and hemorrhage^22–24^. Lastly, the edematous “high FLAIR” tissuetype was further sub-classified into Edema1 (“tumor-associated”) or Edema2 (“leukoaraiosis”) on the basis of observations in our development data that areas resembling leukoaraiosis that were far from the tumor were slightly hyperintense on unenhanced T1w compared with edematous regions that were adjacent to contrast-enhancing tumor. We explored the hypothesis that intratumoral “high FLAIR” tissue that shares mpMRI characteristics with leukoaraiosis has a different relevance to disease progression than the remainder of the intratumoral “high FLAIR” tissue.

### Validation of Tissuetype Segmentation in an External Dataset:

The generalizability of the decision tree in Fig. 2 was investigated on a multi-institutional mix of 3 T and T mpMRI images of preoperative HGG from the BraTS 2020 training dataset^7–9^. Of the 293 HGG samples available in this dataset, 58 were excluded due to missing annotations, ringing artifacts, inhomogeneity artifacts, or mismatch of sequences between T1w and T1wCE; details and an example of excluded samples are provided in supplementary table ST1 and supplementary figure S1. Ground truth volumes of ED, ET, and NCR/NET from the remaining 235 HGG samples were cross-correlated with 5 tumor tissuetypes calculated using a modified decision tree within an “abnormal VOI” (abVOI) comprising the union of ED, ET, and NCR/NET contours; the absence of ADC maps in BraTS data necessitated the omission of a few steps (indicated in Fig. 2) when computing CE, High FLAIR, Fluid, Edema1, and Edema2 volumes.

### Application of Tissuetype Segmentation to Treatment Dataset:

mpMRI images of participants enrolled in the phase I trial NCT02313272 were preprocessed and calibrated as described above. At each scan date for each patient, a loose contour was manually drawn to encompass the pathologic region visible on all applicable slices of the co-registered T1wCE and FLAIR images. All pixels within this VOI classified as BV, CE, Edema1, Edema2, and Fluid tissuetypes were combined into an abVOI, and all pixels of BV tissuetype within this abVOI were re-classified to CE tissuetype for the purposes of comparing the dynamics of changes in volumes of individual tissue types against ground truth progression, where radiologic progression was on RANO criteria as reported previously^12^. Using the rules in the decision tree in Fig. 2, tissuetype maps were computed for all slices of co-registered mpMRI images at each patient scan date.

### Correlation Analysis of Tissuetype Volumes with TTP in Treatment Dataset:

Pearson correlation analysis of the per-timepoint volumes of tissuetypes CE, Edema1, Edema2, High FLAIR (= Edema1 + Edema2), and Fluid within the abVOI, and the treatment duration defined as the number of days elapsed since day 1 of cycle 1 of treatment (t_C1D1), to the time – to – progression (TTP) at each scan date was performed using R software version 4.1.3.

### Multivariable Model of Tissuetype Volumes to TTP in Treatment Dataset:

Volumes of tissuetypes CE, Edema1, Edema2, High FLAIR, and Fluid within the abVOI, and t_C1D1, from 92 post-C1D1 on-study time points, including a few post-progression time points, from 10 participants in Cohort 1 (Fig. 1) were used as independent variables in a process to identify a multivariable model to predict TTP (continuous dependent variable). Model selection and optimization was performed as follows. First, independent variable sets from the 92 time points were randomly split into 10 nearly equally sized segments. Then, candidate models with all possible linear combinations of the independent variables were identified. For each candidate model, 9 segments were used for training and the remaining segment for validation. This process was repeated 10-fold and an average Akaike Information Criterion (AIC) across the 10 cycles was computed for each candidate multiple linear regression model. Of all the candidate models, the one with the lowest AIC was selected as the optimal model.

### Model Accuracy for Predicting TTP in Treatment Dataset:

We evaluated the accuracy of the optimal model for predicting whether progression would occur within *n* days of a given scan date for *n* = 30, 60, and 90. For all patient-timepoints in cohorts 1–4, each point was labeled 1 if the TTP (true or predicted) ≤ *n* and 0 otherwise. Accuracy was calculated as the ratio of the total correctly identified labels to the total number of labels, as in Eq. (2):

2 $$Accuracy_{\text{threshold,cohort}\;}=\frac{\begin{array}{l} \;\\ CorrectlyIDentifiedlabelsforaintervalthresholdinacohort \end{array}}{totalEquationNumberoflabelsinacohort}$$

### Statistical Analysis:

Pearson correlation analysis was used to assess relationships between tissuetype volumes against ground truth tumor segmentations in the BraTS 2020 data, and between tissuetypes and t_C1D1 against TTP in the treatment dataset. A multivariable linear regression using tissuetype volumes as independent variables was developed to model TTP in the treatment dataset. Stepwise variable selection was further used to finalize the model on AIC with 10-fold cross-validation. Statistical analysis used R software version 4.1.3.

## Figures and Tables

**Table 1:** Treatment Dataset Population Demographics

		Bevacizumab naive (n = 24)	Bevacizumab resistant (n = 8)
Age, Years			
	Median (range)	55.5 (22–68)	49..5 (27–61)
Sex, n			
	Male	14	7
	Female	10	1
Histopathologic diagnosis, n			
	Glioblastoma	22	7
	Anaplastic astrocytoma	2	1
MGMT promoter methylation status, n			
	Methylated	12	2
	UnMethylated	11	2
	Unknown	1	4
IDH mutation status, n			
	Mutant	5	2
	Wildtype	19	3
	Unknown	0	3
Restriction prior to study treatment, n			
	yes	12	2
	No	12	6
Enhancing Tumor Volume (cm^3^)			
	Median Range	5.51	15.1
Recurrence (s), n			
	1st	18	0
	2nd	6	7
	3rd	0	1
Steroid use at day 1 of treatment, n			
	yes	5	2
	No	19	6
PD-L1 expression levels			
	< 1%	15	5
	≥ 1%	6	0
	≥ 10%	1	0
	Unknown	3	3

**Table 2:** Treatment Dataset mpMRI Parameters.

	TR (msec)	TE (msec)	FA (degrees)	Resolution (mm^3^)	TI (msec)
FLAIR 1.5 T (3.0 T)	5000 – 12000 (5000 – 11000)	98 – 335 (125 – 389)	98 – 180 (90 – 120)	0.4x0.4x5 – 1x1x1 (0.5x0.5x1)	1800 – 2855 (1800 – 2800)
T1wCE 1.5 T (3.0 T)	222 – 2200 (8 – 2100)	3 – 20 (2 – 10)	8 – 165 (8 – 70)	0.4x0.4x5 – 1x1x5 (0.5x0.5x1 – 0.9x0.9X4)	N/A
T1w 1.5 T (3.0 T)	222 – 2000 (542 – 2300)	5 – 14 (9 – 10)	60 – 180 (70 – 150)	0.4x0.4x5 – 1x1x5 (0.7x0.7x5 – 0.9x0.9x4)	N/A
T2w 1.5 T (3.0 T)	550 – 6280 (3421 – 6460)	20 – 126 (80 – 95)	15 – 180 (90 – 150)	0.6x0.6x4 – 1x1x5 (0.6x0.6x4)	N/A
DWI 1.5 T (3.0 T), b = 0,1000	3800 – 9600 (3541 – 7400)	74 – 113 (57 – 98)	90 (90, 180)	0.6x0.6x5 – 2x2x5 (0.6x0.6x5 – 1.6x1.6x5)	N/A

**Table 3:** Accuracy of Multivariable Model for Predicting Progression *n* Days Prior to Ground Truth, for *n* = 30, 60, and 90 Days, in the Treatment Dataset.

	IDH-wt	IDH-mut	Clinical Progression	Remote recurrence
<30 days	88.16%	88.46%	71.43%	80.00%
<60 days	78.95%	73.08%	71.43%	56.00%
<90 days	73.68%	61.54%	57.14%	40.00%

## Data Availability

De-identified MRI images from this study are available from the corresponding author upon request under an institutional data sharing agreement.
